# Personalized Supplementation Is Associated with Reduced Inflammatory Biomarkers: A 12-Week Observational Study

**DOI:** 10.3390/life15121887

**Published:** 2025-12-10

**Authors:** Eliza Roeth, Madeline Morris, Asher P. Reynolds, Emma M. Reynolds, Reed Hungerford, Eliza J. Livingston, Andrew W. Richardson, Benjamin T. Bikman, Paul R. Reynolds

**Affiliations:** Department of Cell Biology and Physiology, Brigham Young University, Provo, UT 84602, USA

**Keywords:** inflammation, immunity, supplements, personalized

## Abstract

Chronic low-grade inflammation is a central contributor to the development of cardiovascular disease, metabolic dysfunction, autoimmune disorders, and cognitive decline. Blood-based biomarkers, such as C-reactive protein (CRP), ferritin, homocysteine, white blood cell (WBC) count, and anti-thyroid peroxidase (anti-TPO) antibodies enable quantification and monitoring of systemic inflammation over time. We aimed to evaluate the impact of a 12-week personalized, biomarker-guided supplementation program including micronutrients, hormone support, and peptides on inflammatory and immune-related biomarkers across age- and sex-stratified adult cohorts. Participants (n = 48; 8 per group) were stratified by sex and age (40–49, 50–59, 60–69 years) and underwent blood testing at baseline and 12 weeks. Personalized protocols were developed based on individual biomarker profiles and included targeted interventions with vitamin D, omega-3 fatty acids, B vitamins, zinc, selenium, hormone optimization, and other supportive agents. Primary outcomes were percent changes in CRP, ferritin, homocysteine, WBC count, and anti-TPO antibody levels. CRP levels decreased by 33–46% across all groups, with similarly consistent declines in homocysteine (29–37%) and WBC count (22–28%). Ferritin reductions were most notable in men, particularly in older age groups (up to 48%), while anti-TPO antibody levels declined more prominently in women (up to 22%). These changes are consistent with reduced systemic inflammation, improved methylation status, and potential modulation of autoimmune activity. This biomarker-guided, personalized supplementation protocol was associated with clinically meaningful reductions in key markers of inflammation and immune dysregulation. These findings are suggestive of potential efficacy for precision-based health optimization programs and highlight the need for larger randomized controlled trials (RCTs) to confirm causal effects.

## 1. Introduction

Chronic low-grade inflammation is increasingly understood to be a foundational driver of a wide spectrum of modern diseases, including cardiovascular disease, metabolic syndrome, autoimmune conditions, neurodegeneration, and cognitive decline. Unlike acute inflammation, an essential and temporary immune response, low-grade inflammation is persistent, systemic, and often silent. Such chronic inflammation often lacks overt symptoms yet continuously activates the immune system in a way that promotes long-term tissue damage and functional decline. This insidious process, often referred to as “inflammaging,” accelerates biological aging and underlies many chronic disease states observed in midlife and older adults [1].

Extensive research has linked chronic inflammation to key pathophysiological mechanisms in a variety of conditions. In cardiovascular disease, it contributes to endothelial dysfunction, arterial stiffness, and plaque instability [2]. In metabolic disorders, such as obesity and type 2 diabetes, inflammatory cytokines interfere with insulin signaling and lipid metabolism, perpetuating insulin resistance and glycemic dysregulation [3]. Autoimmune diseases, including autoimmune thyroiditis and rheumatoid arthritis (RA), are driven in part by immune dysregulation exacerbated by ongoing inflammatory signaling, though not all are characterized solely by low-grade inflammation; for example, RA can involve systemic chronic hyperinflammation [4,5], while growing evidence suggests that inflammation-related processes contribute to neurodegeneration and early cognitive decline [6].

Crucially, chronic inflammation is not only measurable, but also modifiable. Advances in blood biomarker testing allow for the identification and quantification of inflammation through markers such as high-sensitivity C-reactive protein (CRP), ferritin (when elevated beyond iron storage function), homocysteine, leukocyte differentials, and thyroid autoantibodies including anti-thyroid peroxidase (TPO) antibodies. These biomarkers provide a functional window into systemic inflammation and serve as dynamic indicators of disease risk and therapeutic response [7,8,9].

We have leveraged these insights to synthesize and evaluate a comprehensive, personalized approach to health optimization. Our model is rooted in biomarker-driven care (see Section 2.3 for details on included vitamins, e.g., vitamin D and B vitamins, hormones, and peptides): blood panels guide the design of individualized supplementation regimens aimed at reducing inflammation and improving biological function. These interventions include evidence-based nutraceuticals, hormone replacement therapies (such as testosterone and estradiol optimization), bioactive peptides for tissue repair and immune modulation, and targeted lifestyle guidance. By integrating multiple modalities and retesting at regular intervals, we are able to dynamically adjust treatment strategies based on real-time biological data.

The following report presents biomarker unadjusted outcomes from participants engaged in this personalized anti-inflammatory protocol. We describe changes observed in five key biomarkers of inflammation over a three-month period across distinct age and gender cohorts. These findings suggest associations supporting the potential of a precision health model to reduce inflammation and its downstream consequences through safe, non-pharmaceutical interventions grounded in individual biology.

## 2. Materials and Methods

### 2.1. Study Design

This study was a retrospective, observational, pre-post intervention trial designed to describe changes in a personalized supplementation program on key biomarkers of chronic low-grade inflammation. Participants underwent baseline and follow-up blood testing over a 12-week period. The 12-week duration was selected because it allows sufficient time for detectable changes in inflammatory biomarkers like CRP, ferritin, and homocysteine, based on prior supplementation studies showing meaningful reductions in 8–12 weeks [10,11,12,13], balancing feasibility, adherence, and clinical relevance in an observational setting. The study was conducted through a telehealth-based platform affiliated with Joi Women’s Health and Get Blokes Men’s Health, which provides biomarker-guided interventions in a personalized clinical setting. The reason for the clinical examination protocol (baseline and 12-week blood testing) and administration of dietary supplements was to provide routine, personalized health optimization for patients seeking to reduce chronic low-grade inflammation and associated risks (e.g., cardiovascular, metabolic, autoimmune). This was not an additional research-imposed intervention but standard practice at the clinics, where biomarker results guided targeted supplementation to address individual deficiencies and improve biological function. As an observational study without experimental manipulation, no control group was included, aligning with its descriptive focus on real-world clinical data; interpretations are thus limited to associations rather than causation, with potential influences from placebo effects or unmonitored variables.

### 2.2. Participants

Participants were recruited from the clinic’s existing patient population and stratified into six demographic subgroups: men and women aged 40–49, 50–59, and 60–69 years, with n = 8 individuals per group. This age range (40–69) was selected to focus on midlife and older adults where chronic low-grade inflammation (‘inflammaging’) accelerates biological aging and disease risk [14,15,16], with stratification by decade (40–49, 50–59, 60–69) to assess age-specific patterns. Eligible participants were adults aged 40 to 69 years with no acute illness at baseline and no recent history of infection, immunosuppressive therapy, or major medical interventions. All data used in this study were obtained as part of routine clinical care. The retrospective analysis was conducted using de-identified data and did not involve any direct patient contact or intervention. As such, specific informed consent for inclusion in this analysis was not required, and the study was determined to be exempt from formal institutional review board (IRB) review under applicable guidelines for quality improvement and retrospective research. Individuals with active autoimmune or inflammatory diseases requiring pharmaceutical treatment were excluded. Patients were observed not for specific diseases but for general health optimization to address chronic low-grade inflammation through personalized programs. No exclusions were made for conditions such as diabetes mellitus, metabolic syndrome, or morbid obesity, provided they met general criteria (e.g., no acute illness or pharmaceutical treatment for active inflammatory/autoimmune diseases). This allowed inclusion of individuals with potential metabolic-related inflammation variants.

### 2.3. Intervention

Participants received interventions as part of routine clinical care, with the primary reason being to improve health through evidence-based, biomarker-driven support, enabling safe reductions in systemic inflammation without pharmaceuticals. All participants received a personalized health plan based on their baseline blood biomarker results. This personalization relied on identified patient data, as standard practice. Personalization was guided by specific thresholds: for instance, the administration of specific micronutrients, such as vitamin D, zinc, and selenium, was guided by baseline deficiency measurements: for example, vitamin D was supplemented if serum 25(OH)D < 30 ng/mL, zinc if plasma zinc < 80 µg/dL, and selenium if levels < 120 µg/L, ensuring targeted correction to support immune modulation (e.g., elevated anti-TPO > 35 IU/mL). Supplements and therapies were tailored to each participant’s biology and monitored through follow-up lab testing and virtual consultations. Dosing varied according to clinical judgment based on age, sex, baseline levels, and response, with typical ranges as follows: vitamin D 1000–5000 IU/day orally, omega-3 1–3 g/day EPA + DHA, B vitamins (B6 10–50 mg, B12 500–1000 µg, folate 400–800 µg daily). Adherence was encouraged via digital support tools and scheduled follow-up visits. Interventions were administered at frequencies typical for clinical practice, such as daily oral intake for micronutrients (e.g., vitamin D, B vitamins) and weekly for hormonal therapies or bioactive peptides. Active peptides included BPC-157 (promotes tissue repair and anti-inflammatory effects via upregulation of growth factors and angiogenesis) and Thymosin Alpha-1 (modulates immunity by enhancing T-cell differentiation and cytokine balance), administered selectively where inflammation or immune dysregulation was indicated, not universally. Distributions varied by subgroup, with hormonal optimization more common in older age groups (e.g., higher prevalence in 60–69 years due to age-related declines) and micronutrients like omega-3 nearly universal across cohorts. Personalization was guided by standard clinical thresholds for deficiencies (e.g., low vitamin D or B-vitamin status per guidelines), without standardized dosing beyond therapeutic norms, to align with individual biology.

### 2.4. Biomarker Assessment

Blood samples were collected at two time points: baseline and approximately 12 weeks after initiating the supplement protocol. All samples were assessed at CLIA-certified laboratories using standardized clinical assays including LabCorp (Burlington, NC, USA), Quest Diagnostics (Secaucus, NJ, USA), and Bioreference Health (Elmwood Park, NJ, USA). Samples were obtained via standard venipuncture. Conditions followed CLIA guidelines: for CRP, ferritin, homocysteine, anti-TPO: serum was collected in red-top/SST tubes, clotted 30–60 min, centrifuged 10–15 min at 1000–2000 g, separated serum was refrigerated 2–8 °C if not immediately analyzed, or stable 14 days RT/ref/frozen. For total WBC counts, whole blood was collected in lavender EDTA tubes, inverted 8–10 times, analyzed within 24–48 h. Transport was conducted at ambient or cold pack to prevent hemolysis/lipemia; rejection for gross hemolysis, or clotting. Inflammatory and immune-related biomarkers were selected as primary outcomes based on their established role in systemic inflammation and disease risk. These included: (1) High-sensitivity C-reactive protein (CRP), measured by immunoturbidimetric assay, (2) Serum ferritin, assessed via enzyme-linked immunosorbent assay (ELISA), (3) Plasma homocysteine, measured using chemiluminescent or mass spectrometry-based methods, (4) Total white blood cell (WBC) count, determined via automated hematology analyzer, reflecting the entire white blood cell population without specific differentials (e.g., neutrophils, lymphocytes, monocytes) as the focus was on total count as a general indicator of immune activation, and (5) Anti-thyroid peroxidase (anti-TPO) antibodies, measured via immunoassay. These biomarkers were selected for their reproducibility, clinical relevance, and responsiveness to lifestyle and nutritional interventions. Additional iron indices were included in comprehensive panels to monitor safety, including hemoglobin (via automated hematology, normal >13 g/dL men, >12 g/dL women) and transferrin saturation (calculated from serum iron and total iron-binding capacity, normal 20–50%), ensuring reductions in ferritin did not indicate deficiency or anemia. Results were aggregated by subgroup (age and sex) for analysis.

### 2.5. Data Analysis

Primary outcomes were defined as changes in biomarker concentrations from baseline to the 12-week follow-up. Data were analyzed using descriptive statistics to calculate mean values and percent changes within each subgroup. Descriptive statistics including the means (averages) were calculated for each biomarker, stratified by sex and age group (40–49, 50–59, and 60–69 years). For each participant, pre- and post-intervention values were collected at baseline and after 12 weeks of a personalized supplementation protocol. Within-group changes in biomarker levels were assessed using paired two-tailed *t*-tests. Normality of the differences between paired observations was assumed due to symmetrical data distribution and verified with visual inspection of residuals. Due to the exploratory nature, small sample size (n = 8 per subgroup), and high number of comparisons (30 *t*-tests: 5 biomarkers × 6 subgroups), no adjustments for multiple comparisons were made originally. However, this increased Type I error risk substantially (FWER ≈ 78.5% for ≥1 false positive). A Bonferroni correction (α = 0.05/30 ≈ 0.0017) would render all *p*-values non-significant. To address this serious flaw and ensure soundness, *p*-values are now removed entirely, with results re-presented as purely descriptive (means, percent changes). Analyses remain paired differences for within-group changes, but interpretations focus on patterns and magnitudes as hypothesis-generating, not inferential. Analyses were conducted separately for each biomarker and demographic group. Statistical analyses were performed with GraphPad Prism 8.0 software.

### 2.6. Ethical Considerations

This study was conducted as part of a clinical quality improvement initiative within a personalized health clinic and did not involve experimental pharmaceuticals. As such, it was exempt from institutional review board (IRB) oversight under prevailing clinical practice guidelines. The retrospective analysis was conducted using de-identified data and did not involve any direct patient contact or intervention. While personalized plans were developed with identified data during clinical care, the retrospective analysis used only de-identified aggregates (e.g., no names, IDs, or traceable details), complying with HIPAA de-identification standards (e.g., removal of 18 identifiers) and qualifying for IRB exemption under 45 CFR 46.104(d)(4) for secondary research on existing data. Accordingly, specific informed consent for inclusion in this analysis was not required. Furthermore, data were de-identified and managed securely in accordance with HIPAA compliance standards.

## 3. Results

Following a 12-week personalized supplementation protocol, participants across all age and sex groups showed associations with meaningful improvements in key biomarkers associated with systemic inflammation, immune regulation, and cardiometabolic risk. Table 1 and Table 2 summarize the mean baseline values, 3-month follow-up mean values, and percentage changes for five primary biomarkers: C-reactive protein (CRP), ferritin, homocysteine, white blood cell (WBC) count, and anti-thyroid peroxidase (anti-TPO) antibodies. Notably, using CRP > 3 mg/L as a criterion for low-grade inflammation, 100% of groups (6/6) had baseline mean CRP above this threshold (3.5–4.6 mg/L), while 100% shifted below (1.9–2.9 mg/L) at 12 weeks, indicating a 100% change in groups meeting the criterion.

### 3.1. Men

Among men, unadjusted reductions in inflammatory biomarkers were observed consistently across all age groups (note: these do not survive multiple comparison corrections; see Methods). CRP, a sensitive indicator of systemic inflammation and cardiovascular risk, declined by 33% in men aged 40–49, 43% in men aged 50–59, and 44% in men aged 60–69. Improvements are consistent with an anti-inflammatory response to the multi-modal intervention. Serum ferritin, which can serve as both an iron and inflammation marker, decreased substantially in the older two male cohorts (−48% in men 50–59 and −41% in men 60–69), with a smaller but notable decline in younger men (−27%). These changes occurred without indications of iron deficiency, as confirmed by stable hemoglobin and transferrin saturation (see Discussion). Homocysteine, an established biomarker for methylation efficiency and vascular health, decreased across all male cohorts by 29–37%, which may reflect likely improvements in B-vitamin status and methylation function. Total WBC count (without differentials), a general indicator of immune activation, dropped by 26–28% across all age groups, returning toward mid-normal range. Anti-TPO antibody levels, an autoimmune thyroid marker, showed smaller but consistent reductions of 9–14%, suggesting modest improvement in subclinical autoimmune activity.

### 3.2. Women

In women, similar unadjusted patterns emerged, with some important distinctions. CRP levels declined across all three female cohorts, with the most dramatic improvement (−46%) seen in women aged 40–49. This likely reflects responsiveness to stress reduction, hormone balancing, or antioxidant support during perimenopausal transition. Older women also experienced significant CRP reductions (−36% in ages 50–59, −37% in 60–69), aligning with the patterns seen in men. Unlike in men, ferritin levels in women declined only modestly, especially in the youngest cohort (−5%), consistent with the likelihood that ferritin in pre- and peri-menopausal women is more reflective of iron status than inflammatory burden. Homocysteine levels improved robustly in all female age groups, decreasing by 33–35%, again pointing to effective support of B-vitamin pathways. Total WBC counts (without differentials) improved modestly in women, with a 22–23% decline across all groups, suggesting immune normalization without suppression. Notably, women exhibited larger percentage reductions in anti-TPO antibodies (−19% to −22%), consistent with baseline-guided supplementation of vitamin D, zinc, and selenium for deficiencies, which may reflect targeted supplementation of these micronutrients contributing to improved thyroid autoimmunity regulation.

## 4. Discussion

This study suggests that a 12-week personalized supplementation protocol targeting hormonal, micronutrient, and inflammatory pathways may be associated with clinically meaningful improvements in biomarkers associated with chronic low-grade inflammation. The 12-week timeframe, common in similar trials [10,11,12,13], allowed for observable biomarker shifts without extended follow-up. These findings are consistent across both sexes and all examined age groups (40–69 years) and underscore the need for larger randomized controlled trials (RCTs) to establish causality. Notably, while the study targeted general chronic low-grade inflammation (‘inflammaging’), some participants may have had meta-inflammation—a variant driven by metabolic dysfunction in conditions like diabetes, metabolic syndrome, or obesity. These were not excluded, as the protocol aimed to broadly reduce inflammatory burden, but future studies should stratify for such pathologies to distinguish effects.

### 4.1. Improvements in Inflammatory Biomarkers

Reductions in C-reactive protein (CRP) were among the most consistent and substantial associations across all cohorts, with relative decreases ranging from 33% to 46% over the 12-week intervention. Regarding the common criterion of CRP > 3 mg/L for low-grade inflammation, this changed by 100%: all group means exceeded it at baseline but fell below post-intervention, aligning with the 33–46% decreases and suggesting widespread resolution. CRP is a hepatic acute-phase reactant that is highly sensitive to systemic inflammatory signaling, particularly via interleukin-6 (IL-6), and has emerged as a robust biomarker of low-grade, chronic inflammation. Elevated CRP, even within high-normal ranges, has been independently linked to increased risk of cardiovascular events, type 2 diabetes, and all-cause mortality, making it a clinically actionable marker even in asymptomatic individuals [17]. Importantly, CRP is also dynamic and modifiable: its levels can respond rapidly to both acute changes in inflammatory burden and to therapeutic interventions. The magnitude of CRP reductions observed in this study is comparable to what has been reported in clinical trials of anti-inflammatory agents, including high-dose omega-3 fatty acids, curcumin, and CoQ10 supplementation [18].

Changes in ferritin levels appeared to be more pronounced in men, particularly in the older age groups, consistent with ferritin’s dual biological role as both an iron storage protein and an acute-phase reactant. Importantly, these reductions were monitored alongside other iron indices to rule out deficiency. Post-intervention hemoglobin levels remained within normal ranges (mean >13.5 g/dL across male groups, no cases. In the context of chronic low-grade inflammation, ferritin levels can become elevated independent of iron status due to upregulation by pro-inflammatory cytokines such as IL-6. Elevated ferritin has been strongly associated with insulin resistance, central obesity, hepatic steatosis, and increased cardiometabolic risk, making it a useful surrogate marker for subclinical inflammation in population-level studies [19]. The larger relative reductions in ferritin among men in this cohort likely reflect a combination of higher baseline values—due in part to sex-related differences in iron turnover and inflammation—and a lower risk of latent iron deficiency compared to women, particularly those of pre- or peri-menopausal status.

Homocysteine levels declined consistently across all demographic groups in this study, with reductions ranging from 29% to 37%. These changes may reflect potential improvements in methylation efficiency and likely B-vitamin status, particularly folate, B6, and B12, which are key cofactors in homocysteine metabolism. Elevated homocysteine, termed hyperhomocysteinemia, is a well-established risk factor for endothelial dysfunction and cardiovascular disease. Mechanistically, homocysteine contributes to vascular damage through multiple pathways, including oxidative stress, nitric oxide depletion, endoplasmic reticulum stress, and pro-inflammatory cytokine signaling [20]. These processes result in impaired vasodilation, increased vascular stiffness, and a pro-atherogenic endothelial phenotype. Further, homocysteine stimulates NADPH oxidase activity, leading to the generation of superoxide radicals and peroxynitrite, which in turn drive oxidative damage to vascular tissues and promote low-grade chronic inflammation [21]. These effects have been observed both in animal models and in endothelial cell cultures. Newer evidence also shows that moderate elevations in homocysteine, more commonly seen in aging adults, can impair endothelial cell migration and energy metabolism through sub-lethal ER stress and mitochondrial dysfunction [22]. Thus, reductions in homocysteine levels appear protective against oxidative damage and the resulting impairment of cellular function that causes widespread inflammation.

Decreases in white blood cell (WBC) count, while moderate (22–28%), appeared biologically meaningful and clinically relevant. These reductions pertain to the total WBC population, as differentials (e.g., neutrophils for acute inflammation or lymphocytes for adaptive immunity) were not separately analyzed. The total count’s prognostic value, as established in large cohorts [9,15,16,17], supports its use here as a marker of overall immune normalization and reduced vascular risk, though subset-specific changes could provide additional mechanistic insights. Elevated WBC count, even within the high-normal range, is a well-established predictor of cardiovascular events, metabolic syndrome, and all-cause mortality. In a large cohort of patients with coronary heart disease, those in the highest tertile of WBC count had a 47% increased risk of total mortality over six years, with each 1000/μL increase in WBC count associated with a 6% rise in mortality Risk [17,23]. Similarly, the Women’s Health Initiative found that postmenopausal women with WBC counts in the upper quartile had more than twice the risk of death from coronary heart disease compared to those in the lowest quartile, independent of CRP levels and traditional risk factors [24]. Beyond its prognostic value, elevated WBC count also reflects ongoing immune activation and low-grade inflammation, which contributes to vascular dysfunction, oxidative stress, and endothelial damage. Mechanistically, increased leukocyte levels, particularly neutrophils, can infiltrate arterial walls, release reactive oxygen species, and trigger cytokine cascades that accelerate atherosclerosis [25]. Decreased WBC counts demonstrate plausible systemic inflammation improvements and the preservation of tissue function, especially evident in cardiac and vascular tissue.

Lastly, we assessed anti-thyroid peroxidase (anti-TPO) antibodies, which are autoimmune markers commonly elevated in subclinical hypothyroidism and Hashimoto’s thyroiditis. Anti-TPO declined in both sexes over the 12-week period, with more pronounced reductions observed in women. Anti-TPO antibodies target thyroid peroxidase, a key enzyme in thyroid hormone synthesis, and are highly predictive of future thyroid dysfunction even in euthyroid individuals. Their presence is associated not only with autoimmune thyroid disease but also with systemic inflammatory burden and metabolic dysregulation, particularly in women over 40. However, as noted in RA, autoimmune inflammation can range from low-grade to hyperinflammation [20], though our study exclusions for active pharmaceutical-requiring cases focused on subclinical, low-grade presentations to target modifiable chronic burden. Although anti-TPO levels are more stable and less reactive to short-term influences compared to acute-phase markers like CRP, growing evidence suggests they can be modulated through targeted nutritional and immunological interventions. The observed reductions in this study may suggest improved immune tolerance and a dampening of autoimmune signaling pathways, potentially mediated by optimized levels of vitamin D, zinc, and selenium: three micronutrients known to influence T-helper cell balance and reduce thyroid-specific autoimmunity. This administration was specifically guided by baseline deficiency measurements, as detailed in Methods, confirming corrections were applied where indicated (e.g., in cases of vitamin D insufficiency common in women over 40). Selenium, in particular, has been shown in multiple trials to reduce anti-TPO titers by 20–40% over a 3–6 month period in patients with Hashimoto’s thyroiditis [26]. Vitamin D deficiency has also been linked to increased prevalence and severity of autoimmune thyroid disease, and its correction may contribute to immune modulation [9].

### 4.2. The Role of Personalized Supplementation

A key differentiator in this program is the precision, biomarker-guided approach to supplement selection. Unlike generic supplement plans, this model targets underlying biological dysfunction by aligning intervention with real-time physiology. The emerging field of precision nutrition supports this methodology. In a recent trial, participants receiving genetically and phenotypically tailored dietary plans demonstrated significantly greater reductions in CRP, TNF-α, and other inflammatory biomarkers compared to those on standard diets [18]. Further, a recent review concluded that biomarker-guided dietary supplementation enables more effective, individualized therapeutic interventions that are responsive to immune, hormonal, and metabolic profiles, thereby enhancing both efficacy and safety [27]. This biomarker-guided approach allowed for transparent tailoring, with decisions based on predefined thresholds as detailed in Methods. These correlative findings suggest that chronic inflammation is not only measurable but also modifiable, and that real-time monitoring combined with personalized protocols may create biologically meaningful changes, thereby justifying larger controlled trials (RCTs) to evaluate true effectiveness.

### 4.3. New Developments, Limitations, and Future Prospects

Recent developments in personalized supplementation highlight innovative approaches to reducing chronic low-grade inflammation. For instance, probiotics and synbiotics have shown efficacy in lowering pro-inflammatory cytokines in prediabetes and type 2 diabetes, with a 2023 randomized trial demonstrating significant IL-6 and TNF-α reductions over 12 weeks [28]. Cocoa extract supplementation in older adults has similarly decreased hsCRP by 15–20% in a 2024 study, attributing effects to flavonoid-mediated antioxidant activity [29]. Advances in AI and genetic-guided nutrition enable more precise interventions, as evidenced by 2024 reviews on biomarker-driven dietary strategies that optimize immune and metabolic profiles for enhanced safety and efficacy [30,31]. Omega-3/n-6 fatty acid ratios continue to be refined, with 2025 meta-analyses confirming anti-inflammatory benefits in diverse populations [18,32]. These innovations build on traditional micronutrient therapies, incorporating multimodal elements like peptides for targeted immune modulation [33].

Although this study observed promising biomarker improvements, several limitations must be acknowledged. As a retrospective, observational pre-post design intended for descriptive, hypothesis-generating purposes, a control group was not incorporated, which is consistent with such non-experimental studies; however, this absence limits causal inference and the ability to rule out confounders such as placebo effects, regression to the mean (especially in participants seeking intervention due to elevated baseline values), or unmeasured lifestyle changes (e.g., diet, exercise, or stress reduction) that are often encouraged in personalized health programs. The small sample size (n = 8 per subgroup) further reduces generalizability and statistical power. Furthermore, the 30 unadjusted *t*-tests inflate Type I error risk, with a ~78.5% FWER for at least one false positive. While a Bonferroni correction (α ≈ 0.0017) would render all findings non-significant, the consistent directional reductions across all biomarkers and subgroups (e.g., CRP decreased 33–46% in every group) suggest patterns unlikely due solely to chance, supporting hypothesis generation. Additionally, the original unadjusted *p*-values constituted a serious statistical flaw, undermining soundness due to high multiplicity (30 tests) and small n, with unreliable inference (e.g., Bonferroni would nullify significance). Results are now purely exploratory and descriptive, with *p*-values removed to avoid misleading claims; interpretations rely on consistent reduction patterns (e.g., all biomarkers decreased 22–48% across groups) as suggestive signals for future confirmatory RCTs with adjustments. Participants were drawn from a specific clinical population, potentially introducing selection bias toward motivated individuals. Adherence was encouraged but not quantitatively assessed, and the study did not account for demographic diversity (e.g., race/ethnicity) or long-term outcomes beyond 12 weeks. Overall, the findings of this study are correlative and suggestive of potential effectiveness rather than definitive proof of causality. They primarily serve to justify the pursuit of future, larger randomized controlled trials (RCTs) to rigorously test the protocol’s impact. While aggregate details on interventions and baselines are now provided to address the ‘black box’ nature, full replicability is limited by the personalized, clinical context. Additionally, reliance on total WBC count without leukocyte differentials (e.g., neutrophils/lymphocytes) limits clarity on which cell populations drove reductions, potentially overlooking subset-specific effects; future studies should include differentials for more granular analysis. Finally, the lack of exclusion or stratification for diabetes mellitus, metabolic syndrome, or morbid obesity means meta-inflammation (metabolically-triggered low-grade inflammation) could have influenced results in some participants, potentially confounding general inflammaging interpretations; confirmatory studies should address this through subgroup analysis.

Future prospects include larger randomized controlled trials (RCTs) with control arms (e.g., placebo, non-personalized supplements) to establish causality, long-term follow-up beyond 12 weeks to assess sustained effects, and diverse cohorts incorporating racial/ethnic and metabolic stratification. Integration of emerging technologies like AI for real-time personalization, genomics for tailored nutrient responses, and advanced biomarkers (e.g., cytokine panels) could further refine protocols [34,35,36]. Additionally, exploring combinations with lifestyle interventions or novel agents (e.g., probiotics, cocoa derivatives) may enhance outcomes in specific subpopulations, advancing precision health optimization.

## Figures and Tables

**Table 1:** Three-Month Biomarker Improvements in Men.

Group	Marker	Baseline Avg.	3-Month Avg.	% Change
**Men 40–49**	CRP (mg/L)	3.9	2.6	−33%
	Ferritin (ng/mL)	290	212	−27%
	Homocysteine (mmol/L)	13.2	9.4	−29%
	WBC (×10^9^/L)	9.8	7.1	−28%
	Anti-TPO Ab (IU/mL)	185	169	−9%
**Men 50–59**	CRP (mg/L)	4.4	2.5	−43%
	Ferritin (ng/mL)	340	177	−48%
	Homocysteine (mmol/L)	14.8	9.3	−37%
	WBC (×10^9^/L)	10.1	7.5	−26%
	Anti-TPO Ab (IU/mL)	195	170	−13%
**Men 60–69**	CRP (mg/L)	5.2	2.9	−44%
	Ferritin (ng/mL)	375	221	−41%
	Homocysteine (mmol/L)	15.2	9.8	−36%
	WBC (×10^9^/L)	10.4	7.6	−27%
	Anti-TPO Ab (IU/mL)	210	180	−14%

*p*-values are not presented due to unreliability from multiple comparisons; applying Bonferroni correction (α = 0.0017 for 30 tests) would result in no significant findings.

**Table 2:** Three-Month Biomarker Improvements in Women.

Group	Marker	Baseline Avg.	3-Month Avg.	% Change
**Women 40–49**	CRP (mg/L)	3.5	1.9	−46%
	Ferritin (ng/mL	110	105	−5%
	Homocysteine (mmol/L)	12	8.1	−33%
	WBC (×10^9^/L)	9	7	−22%
	Anti-TPO Ab (IU/mL)	310	250	−19%
**Women 50–59**	CRP (mg/L)	4.2	2.7	−36%
	Ferritin (ng/mL	150	125	−17%
	Homocysteine (mmol/L)	13.1	8.5	−35%
	WBC (×10^9^/L)	9.2	7.2	−22%
	Anti-TPO Ab (IU/mL)	330	260	−21%
**Women 60–69**	CRP (mg/L)	4.6	2.9	−37%
	Ferritin (ng/mL	160	130	−19%
	Homocysteine (mmol/L)	13.9	9.2	−34%
	WBC (×10^9^/L)	9.5	7.3	−23%
	Anti-TPO Ab (IU/mL)	360	280	−22%

*p*-values are not presented due to unreliability from multiple comparisons; applying Bonferroni correction (α = 0.0017 for 30 tests) would result in no significant findings.

## Data Availability

Data and other materials are available from the corresponding author on reasonable request.

## Abbreviations

The following abbreviations are used in this manuscript:

CRP	C-reactive protein
WBC	White blood cell
TPO	Thyroid peroxidase
ELISA	Enzyme linked immunosorbent assay

## References

[1] Kipinoinen T., Toppala S., Rinne J.O., Viitanen M.H., Jula A.M., Ekblad L.L. (2022). Association of Midlife Inflammatory Markers With Cognitive Performance at 10-Year Follow-up. Neurology.

[2] Jeppesen J. (2011). Low-grade chronic inflammation and vascular damage in patients with rheumatoid arthritis: Don’t forget “metabolic inflammation”. J. Rheumatol..

[3] Esposito K., Giugliano D. (2004). The metabolic syndrome and inflammation: Association or causation?. Nutr. Metab. Cardiovasc. Dis..

[4] Zotova N., Zhuravleva Y., Chereshnev V., Gusev E. (2023). Acute and Chronic Systemic Inflammation: Features and Differences in the Pathogenesis, and Integral Criteria for Verification and Differentiation. Int. J. Mol. Sci..

[5] Del Pinto R., Ferri C. (2018). Inflammation-Accelerated Senescence and the Cardiovascular System: Mechanisms and Perspectives. Int. J. Mol. Sci..

[6] Lasselin J., Capuron L. (2014). Chronic low-grade inflammation in metabolic disorders: Relevance for behavioral symptoms. Neuroimmunomodulation.

[7] Gilbert-Diamond D., Baylin A., Mora-Plazas M., Villamor E. (2012). Chronic inflammation is associated with overweight in Colombian school children. Nutr. Metab. Cardiovasc. Dis..

[8] Cramer D.W., Vitonis A.F. (2017). Signatures of reproductive events on blood counts and biomarkers of inflammation: Implications for chronic disease risk. PLoS ONE.

[9] Haghbin M., Razmjooei F., Abbasi F., Rouhie R., Pourabbas P., Mir H., Roustazadeh A., Mofazzal Jahromi M.A., Bagheri K. (2024). Evaluation of the hematological parameters, inflammatory biomarkers, and thyroid hormones in hypothyroidism patients. BMC Res. Notes.

[10] Walentukiewicz A., Lysak-Radomska A., Jaworska J., Prusik K., Prusik K., Kortas J.A., Lipiński M., Babinska A., Antosiewicz J., Ziemann E. (2018). Vitamin D Supplementation and Nordic Walking Training Decreases Serum Homocysteine and Ferritin in Elderly Women. Int. J. Environ. Res. Public Health.

[11] Pereira S.L., Shoemaker M.E., Gawel S., Davis G.J., Luo M., Mustad V.A., Cramer J.T. (2022). Biomarker Changes in Response to a 12-Week Supplementation of an Oral Nutritional Supplement Enriched with Protein, Vitamin D and HMB in Malnourished Community Dwelling Older Adults with Sarcopenia. Nutrients.

[12] Ooi T.C., Ahmad A., Rajab N.F., Sharif R. (2023). The Effects of 12 Weeks Colostrum Milk Supplementation on the Expression Levels of Pro-Inflammatory Mediators and Metabolic Changes among Older Adults: Findings from the Biomarkers and Untargeted Metabolomic Analysis. Nutrients.

[13] Sun Y. (2025). Effects of 12-Week Multiple Micronutrient Supplementation on Immune Function, Body Weight, and Metabolic Parameters in Middle-Aged Women: A Retrospective Study. Cureus.

[14] Ferrucci L., Fabbri E. (2018). Inflammageing: Chronic inflammation in ageing, cardiovascular disease, and frailty. Nat. Rev. Cardiol..

[15] Furman D., Campisi J., Verdin E., Carrera-Bastos P., Targ S., Franceschi C., Ferrucci L., Gilroy D.W., Fasano A., Miller G.W. (2019). Chronic inflammation in the etiology of disease across the life span. Nat. Med..

[16] Franceschi C., Campisi J. (2014). Chronic inflammation (inflammaging) and its potential contribution to age-associated diseases. J. Gerontol. A Biol. Sci. Med. Sci..

[17] Zakai N.A., Katz R., Jenny N.S., Psaty B.M., Reiner A.P., Schwartz S.M., Cushman M. (2007). Inflammation and hemostasis biomarkers and cardiovascular risk in the elderly: The Cardiovascular Health Study. J. Thromb. Haemost..

[18] Kavyani Z., Musazadeh V., Fathi S., Hossein Faghfouri A., Dehghan P., Sarmadi B. (2022). Efficacy of the omega-3 fatty acids supplementation on inflammatory biomarkers: An umbrella meta-analysis. Int. Immunopharmacol..

[19] Shim Y.S., Kang M.J., Oh Y.J., Baek J.W., Yang S., Hwang I.T. (2017). Association of serum ferritin with insulin resistance, abdominal obesity, and metabolic syndrome in Korean adolescent and adults: The Korean National Health and Nutrition Examination Survey, 2008 to 2011. Medicine.

[20] Lai W.K., Kan M.Y. (2015). Homocysteine-Induced Endothelial Dysfunction. Ann. Nutr. Metab..

[21] Edirimanne V.E., Woo C.W., Siow Y.L., Pierce G.N., Xie J.Y., O K. (2007). Homocysteine stimulates NADPH oxidase-mediated superoxide production leading to endothelial dysfunction in rats. Can. J. Physiol. Pharmacol..

[22] Chatterjee B., Fatima F., Seth S., Sinha Roy S. (2024). Moderate Elevation of Homocysteine Induces Endothelial Dysfunction through Adaptive UPR Activation and Metabolic Rewiring. Cells.

[23] Haim M., Boyko V., Goldbourt U., Battler A., Behar S. (2004). Predictive value of elevated white blood cell count in patients with preexisting coronary heart disease: The Bezafibrate Infarction Prevention Study. Arch. Intern. Med..

[24] Margolis K.L., Manson J.E., Greenland P., Rodabough R.J., Bray P.F., Safford M., Grimm R.H., Howard B.V., Assaf A.R., Prentice R. (2005). Leukocyte count as a predictor of cardiovascular events and mortality in postmenopausal women: The Women’s Health Initiative Observational Study. Arch. Intern. Med..

[25] Welsh C., Welsh P., Mark P.B., Celis-Morales C.A., Lewsey J., Gray S.R., Lyall D.M., Iliodromiti S., Gill J.M.R., Pell J. (2018). Association of Total and Differential Leukocyte Counts With Cardiovascular Disease and Mortality in the UK Biobank. Arter. Thromb. Vasc. Biol..

[26] Lin Z., Lim S., Lim M.S. (2003). Growth regulation by p27Kip1 is abrogated by multiple mechanisms in aggressive malignant lymphomas. Br. J. Haematol..

[27] Pokushalov E., Ponomarenko A., Shrainer E., Kudlay D., Miller R. (2024). Biomarker-Guided Dietary Supplementation: A Narrative Review of Precision in Personalized Nutrition. Nutrients.

[28] Baroni I., Fabrizi D., Luciani M., Magon A., Conte G., De Angeli G., Paglione G., Ausili D., Caruso R. (2024). Probiotics and synbiotics for glycemic control in diabetes: A systematic review and meta-analysis of randomized controlled trials. Clin. Nutr..

[29] Li S., Hamaya R., Zhu H., Clar A., Rist P.M., Huang Y., Manson J.E., Sesso H.D., Dong Y. (2025). Effects of 2-year cocoa extract supplementation on inflammaging biomarkers in older US adults: Findings from the COcoa Supplement and Multivitamin Outcomes Study randomised clinical trial. Age Ageing.

[30] Wang X., Sun Z., Xue H., An R. (2025). Artificial Intelligence Applications to Personalized Dietary Recommendations: A Systematic Review. Healthcare.

[31] Wu X., Oniani D., Shao Z., Arciero P., Sivarajkumar S., Hilsman J., Mohr A.E., Ibe S., Moharir M., Li L.J. (2025). A Scoping Review of Artificial Intelligence for Precision Nutrition. Adv. Nutr..

[32] Gubari M.I.M. (2024). Effect of omega-3 fatty acid supplementation on markers of inflammation and endothelial function in patients with chronic heart disease: A systematic review and meta-analysis. Cell. Mol. Biol..

[33] Liu H., Zhang L., Yu J., Shao S. (2024). Advances in the application and mechanism of bioactive peptides in the treatment of inflammation. Front. Immunol..

[34] McFarlin B.K., Vingren J.L., Hill D.W., Bridgeman E.A. (2023). MSM Supplementation Is Associated with Reduced Inflammation and Improved Innate Immune Response following In Vitro LPS-Stimulation in Humans after a Bout of Downhill Running. Muscles.

[35] Singar S., Nagpal R., Arjmandi B.H., Akhavan N.S. (2024). Personalized Nutrition: Tailoring Dietary Recommendations through Genetic Insights. Nutrients.

[36] Agrawal K., Goktas P., Kumar N., Leung M.F. (2025). Artificial intelligence in personalized nutrition and food manufacturing: A comprehensive review of methods, applications, and future directions. Front. Nutr..

